# Seroprevalence and associated risk factors of *Toxoplasma gondii* among pregnant women in Ain Defla, northwest Algeria

**DOI:** 10.4314/ahs.v25i1.7

**Published:** 2025-03

**Authors:** Asma Dahmani, Asma Aiza, Safia Zenia

**Affiliations:** 1 Institute of Veterinary Sciences, University Blida1, B.P. 270, Road of Soumaa, 09000, Blida, Algeria; 2 Faculty of Natural and Life Sciences and Earth Sciences-Djilali Bounaama University of Khemis Miliana, Algeria; 3 Laboratory research «Animal Health and Production» Superior National Veterinary School - B.P. 165, Issad Abbas, El Alia. Algiers, Algeria

**Keywords:** *T.gondii* infection, Pregnancy, Seroprevalence, Antibodies, Risk Factors

## Abstract

**Background:**

*Toxoplasma gondii* (*T. gondii*) is a serious public health issue, but limited data has been published to date on the seroprevalence of *T. gondii* infection in Algeria.

**Objectives:**

The aim of this study was to determine the seroprevalence and associated risk factors of *T.gondii* infection among pregnant women in Ain Defla, northwest Algeria.

**Methods:**

Blood samples were collected from 300 pregnant women. Serum samples were analyzed to detect *T. gondii* antibodies (immunoglobulin M and immunoglobulin G) via the enzyme-linked immunosorbent assay (ELISA) technique. Risk factors associated with *T.gondii* infection was assessed through structured questionnaire.

**Results:**

The overall seroprevalence of *T.gondii* infection was 32.03%. Seropositivity for IgG only was 22.78%, IgM only was 2.14% and cooccurrence of IgG/IgM was 7.12%. Pregnant women who lived in rural areas (OR= 1.739; 95%CI: 1.009–2.997; P=0.045), eat raw vegetables (OR=2.659; 95%CI: 1.575–4.488; P<0.0001), who drunk tap water (OR=3.806; 95%CI: 2.248–6.442; P<0.0001), were in contact with soil (OR= 2.836; 95%CI: 1.620- 4.967; P<0.0001) and have not meals at home (OR=3.221; 95%CI: 1.864 – 5.565; P<0.0001) were significantly at risk of infection with *T. gondii*.

**Conclusion:**

Our findings emphasize the need for higher awareness of *T. gondii* infection in Algeria. Public education and serological screening programm should be recommended.

## Introduction

Toxoplasmosis is considered as one of the main infectious agents during pregnancy^1^. It is mainly acquired by eating raw or undercooked meat that contains *T. gondii* tissue cysts, by ingesting oocysts shed by cats from soil, by drinking contaminated water, and by vertical transmission from mother to fetus through the placenta during pregnancy^2^,^3^,^4^,^5^. The tissue cyst-forming coccidium *T. gondii* is one of the more polyxenous parasites known to date. It has a facultatively heteroxenous life cycle and can probably infect all warm-blooded animals (mammals and birds) and humans^6^. Cats and other members of the Felidae are the definitive hosts of the parasite and shed the oocysts after they are infected. These oocysts contain infective sporozoites that can then cause human infection by fecal-oral transmission. Humans can also be exposed to bradyzoites, contained in tissue cysts of the intermediate hosts—particularly food animals—through consumption of improperly cooked meat and meat products or water^7^. The immunologic response to primary infection is followed by encystment of the parasite (latent toxoplasmosis), providing life-long immunity. Possible reactivation of latent infection in an increasingly immunosuppressed population, however, makes toxoplasmosis an important opportunistic infection^8^. Several factors are associated with *Toxoplasma* prevalence, including low socioeconomic status, owning cats, eating raw or uncooked meat, eating raw or unwashed vegetables or fruits^9^, having poor hand hygiene^10^,^11^ and contact with soil^12^,^13^.

However, to date, the relative effects of each of these factors have not been fully established, except for the fact that they vary from area to area. Although, when maternal infection is acquired during pregnancy, *T. gondii* can infect the fetus with variable severity. However, congenital infection during the first trimester can lead to more severe disease when it occurs^14^. The seroprevalence of *T. gondii* infection is extremely varied greatly among different countries^6^,^15^, among different geographical areas within one country^6^,^16^, and among different ethnic groups living in the same area^6^,^15^,^16^. In Europe seroprevalence of anti-*T.gondii* antibodies in pregnant women vary from 9% to 67%^17^,^18^ and in Asia 0.8% to 63.9% seroprevalence were reported^19^. On top of this in Africa different scholars were reported 18.5% to 92.5% seroprevalence^20^,^21^. However, Robinson et al.^22^ and Mocanu et al.^23^ noted a decrease of the trend of *T.gondii* seroprevalence in the last years as a result of implementing hygiene measures. In Algeria, few studies on the seroprevalence and potential risk factors associated with *T.gondii* infection in pregnant women have been performed. On the other hand, Algerian's humid, and rainy climate is changing and other environmental variables affect the emergence of potential pathogens. In light of these data, we performed a cross-sectional study to determine the prevalence of *T. gondii* infection in pregnant women living in Ain Defla region and the risk factors associated with the seropositivity of *T. gondii*.

## Methods

### Description of study area and targeted population

This cross-sectional study was conducted between January and April of 2022 in the hospital of Meliana and five antenatal (ANC) clinics distributed in the region of Ain Defla. The study population comprised of 300 pregnant women from 1st trimester to their 3rd trimester or at term,. These women visited the hospital and clinics for the purpose of ANC and they were selected by systematic random sampling. Permission for the study was taken from Directorate of Health. The samples were obtained of 281 pregnant women who met the inclusion criteria: to be a pregnant woman, to reside within or around the city of Ain Defla, to have renatal exams, to be aged between 20 and 40 years old, and to accept participating in the research. To select the sample, we used the simple random sampling method, with a systematic step size of around 4, from a total of 1,187 pregnant women attending our clinics. 300 were selected for the survey.

### Questionnaire survey

Data regarding the associated risk factors were collected from all pregnant women during a face-to-face interview using a structured questionnaire by gathering sociodemographic data that included the participant's age, education level, residence, knowledge of transmission modes. In addition, behavioral and lifestyle information were also collected (e,g., consumption of raw or undercooked meat, consumption of raw fruit or vegetables, contact with cats, drinking tap water, exposure to garden soil, eating out, washing vegetables and fruits with water Bleach or vinegar and blood transfusion).

### Serum samples and serological method

Approximately 5 mL sample of venous-blood was aseptically collected from each study participant by venipuncture into a tube. The tubes containing the blood were then labeled and kept it at 4°C before transport to the hostipal and clinical laboratories under suitable conditions. All blood samples were centrifuged at 3000 rpm for 5 minutes, and serum samples were collected and stored at −20°C until analysis. Serum samples were tested using ELISA commercial kits BIO RAD (France) (PLATELIA™ TOXO IgG ref 72840 IVD CE 0459 and PLATELIA™ TOXO IgM ref 72841 IVD CE 0459). We quantitatively evaluated the anti-*T. gondii* IgG antibodies while IgM specific antibodies were qualitatively detected. The steps to test the samples were done according to the manufacturer's instructions. *T. gondii* IgG antibody concentration levels were expressed as international units IU/mL, and a result equal to or greater than 9 IU/mL was considered positive for *T. gondii* and indicated past or chronic, latent infection. *T. gondii* IgG antibody concentration levels < 6 IU/mL were considered negative. The unit for *T. gondii* IgM results was index. The results of IgM antibodies with ≥ 1 index were considered positive for *T. gondii* and indicated a recently acquired infection. The results of IgM antibodies with < 0.80 index were considered negative for *T. gondii*.

### Statistical analysis

For statistical analysis, univariate and multivariate analyses were performed using the software program Microsoft Excel 2010 and Statistical Data for Social Science (SPSS) 20 packages © IBM Copyright, IBM Corporation and its licensors 1989. 2011 IBM., USA. The univariate analysis was carried out to evaluate each independent variable for its unadjusted association with *T.gondii* infection. The Associations among the variables were assessed using crude odds ratios (ORs), and 95% confidence intervals (CIs) were calculated, and Chi-square analyses were performed. All independent variables associated with *T.gondii* infection at p < 20% threshold were included in a multivariate analysis using binary logistic regression to evaluate factors associated with *T.gondii* infection. For the comparison between the two serological tests, the degree of agreement (Cohn's Kappa) was calculated based on contingency tables containing date from both serum panels. Degree of agreement was interpreted as follows: ϰ ≤ 0.2 = slight, 0.21 ≤ ϰ ≤ 0.4 = fair, 0.41 ≤ ϰ ≤ 0.6 = moderate, 0.61 ≤ ϰ ≤ 0.8 = substantial, ϰ>0.8 = almost perfect, ϰ = 1 = perfect^24^.

## Results

### Comparison between IgG and IgM

Table 1 shows that 64 (91.43%) participants have positive IgG antibodies to *T. gondii*, indicating previous infection, and 6 (8.57%) have specific IgM antibodies to *T. gondii*, indicating acute infection. It also shows that 20 of the participants have both acute and chronic infection, indicated by the positive presence of IgM and IgG antibodies. Statistical analysis showed significant differences between them (p<0.0001). In addition, to study the agreement between the two techniques, we will use Cohn's Kappa measure of agreement. The value of the kappa index is 0.259 with 95% CI (0.21 - 0.40), the significance is p-value<0.0001. According to the interpretation of the index, the agreement between the two techniques is equitable.

**Table 1:** Comparison between IgG and IgM

ELISA	N	Number of women tested				
		Positive		Negative		P-value
test		No	%	No.	%	P<0.0001 Test de khi-deux
IgG	70	64	91.43%	6	8.57%	
IgM	70	6	8.57%	64	91.452%	
IgG & IgM	211	20	9.48%	191	90.52%	

### Seroprevalence of *T. gondii* in pregnant women

Among 281 pregnant women serum studied, 90 (32.03%) were found to be seropositives. Seropositivity for *Toxoplasma* IgG only was highest (22.78%, 64/281) classified as latent or chronic infection. Seropositivity for *Toxoplasma* IgM only was 2.14% (6/281) classified as acute infections. while 7.12% (20/281) of samples had both acute and chronic infection which is indicated by the presence positive of both IgM and IgG antibodies.

### Risk factors associated with *T.gondii* infection

Results of the seroprevalence of *T.gondii* according to the risk factors are shown in the following Table:

**Table 2:** Risk factors for *T. gondii*

Variables	N	Seropositive		Seronegative		P	OR	CI 95%
		n	%	n	%			
**Age**								
< 30 years ≥ 30 years	174 107	53 37	30.5 34.6	121 70	69.5 65.4	0.472	0.829 1.207	0.496 – 1.384 0.723 – 2.015
**Education level**								
University Non university	130 151	38 52	29.23 34.43	92 99	70.76 65.56	0.351	0.786 1.272	0.474 – 1.304 0.767 – 2.108
**Area or residence**								
Rural Urban	78 203	32 58	41.02 28.57	46 145	58.97 71.42	0.045	1.739 0.575	1.009 – 2.997 0.334 – 0.991
**Knowledge of women about toxoplasmosis**								
Yes No	198 83	62 28	31.31 33.73	136 55	68.68 66.26	0.691	0.895 1.117	0.519 – 1.545 0.647 – 1.926
**Eat undercooked meat**								
Yes No	44 237	9 81	20.453 4.17	35 156	79.54 65.82	0.073	0.495 2.019	0.227 – 1.081 0.925 – 4.406
**Eating raw vegetables**								
Yes No	142 139	60 30	42.252 1.58	82 109	57.74 78.41	<0.001	2.659 0.376	1.575 – 4.488 0,223 – 0,635
**Contact with cats**								
Yes No	66 215	24 66	36,363 0.69	42 149	63.63 69.30	0.388	1.29 0.775	0.723 – 2.30 0.434 – 1.383
**Tap water consumption**								
Yes No	108 173	54 36	50 2.80	54 137	50 79.19	<0.001	3.806 0.263	2.248 – 6.442 0.155 – 0.445
**Soil contact**								
Yes No	70 211	35 55	50 26.06	35 156	50 73.93	<0.001	2.836 0.365	1.620 – 4.967 0.209 – 0.638
**Blood transfusion**								
Yes No	7 274	4 86	57.143 1.38	3 188	42.85 68.61	0.216 test F	2.915 0.343	0.638 – 13.307 0.075 – 1.566
**Meals at home**								
Yes No	203 78	50 40	24.635 1.28	153 38	75.36 48.71	<0.001	0.310 3.221	0.180 – 0.536 1.864 – 5.565
**Washing vegetables and fruits with bleach or vinegar**								
Yes No	173 108	48 42	27.74 38.88	125 66	72.25 61.11	0.051	0.603 1.657	0.362 – 1.005 0.995 – 2.761

### Multivariates analysis

We decided to continue the analysis using logistic regression, introducing into the model the variables linked to infection (serology) at the p < 20% threshold. The analysis was therefore not continued with the following five variables, which were eliminated at this stage of the bivariate analysis: age, education level, knowledge of transmission modes, contact with cats and blood transfusion. Only 7 variables were entered into the logistic regression model: Region, consumption of undercooked meat, consumption of raw vegetables, consumption of water other than tap water, contact with soil, meals at home and washing of vegetables and fruit with bleach or vinegar. The logistic regression used the input procedure. For each model step, the number of iterations was small (less than 5). The Hosmer-Lemeshow test (goodness-of-fit statistic) of the model (*χ*^2^ = 9.863, dl = 8; p = 0.275) shows that the model fit is adequate (the null hypothesis of a good fit is not rejected, p > 0.05). At the end of the multivariate analysis, the region variable was no longer linked to infection and was eliminated from the model. The final model retained the 5 variables. The variable region, with a p-value of 0.919, and the variable vegetable and fruit washing with bleach or other, with a p-value of 0.636, are not among the variables linked to *T.gondii* infection. It's surprising that these two important variables were eliminated at the end of the analysis. In fact, we can show that the variable region and the variable washing vegetables and fruit with bleach or vinegar are linked to the other variables. The statistical indicator of the overall significance of the model is the measure of association given by the chi-square test, which is worth = 57.529 with ddl=5 and a p-value <0.0001. We can conclude that overall, these 5 variables are significantly associated with *T.gondii* infection. The quality of this regression can be assessed by means of the coefficients of determination proposed by SPSS, namely: Cox & Snell R-two = 0.185 and Nagelkerke R-two=0.2592. This means that between 18.5% and 25.9% of the variability in the probability of being infected with *T.gondii* can be explained collectively by the 5 variables in the model.

### Model accuracy

Table 3 shows a classification table resulting from the prediction model. The model has an accuracy of 71% with an error rate of 29%. It correctly predicts (35 + 164) cases and is wrong in (27+55) cases, giving a ratio of 71% (199/82).

**Table 3:** Classification table resulting from the prediction model

Observations		Forecasts		
		Results *T.gondii* infection		Percent correct
		Positive	Negative	
Results *T.gondii* infection	Positive	35	55	38.9
	Negative	27	164	85.9
Overall percentage				70.8

According to these results, the relevant variables for predicting *T. gondii* infection are: consumption of undercooked meat, consumption of raw vegetables, consumption of water other than tap water, contact with soil, meals at home.

**The equation of the logistic regression model is** Logit (*T.gondii* infection)= -1.956 -1.237* consumption of undercooked meat(Yes)+0.653* consumption of raw vegetables(Yes)+0.933* consumption of water other than tap water(Yes) + 1.360* contact with soil(Yes)+0.763* meals at home(No)

## Discussion

**Overall seroprevalence of** *T. gondii* In the present study the overall seroprevalence of *T.gondii* infection was close to the result of Silva-Díaz et al.^25^ from Peru, Ramsewak et al.^26^ from Trinidad and Tobago and Tavares et al.^27^ from Brazil who reported a seroprevalence of 35.8%, 39.3% and 43.5% respectively. However, it was lower than the 51.5 % seropositive rate reported for Morocco^28^, 75.7% for Ethiopia^29^, 80% for Cameroon^30^. In another hand, the present result was higher than the result reported by different studies in pregnant women from different countries; 20.2% from Sudan^31^, 12.2% from Rwanda^32^, 10 % in China 17 and 11% in Vietnam^33^. Seroprevalence of *T. gondii* infection varies by country, the area of a given country, and the population surveyed. Such variations may be due to the different contexts and impacts of various potential risk factors in these countries. The use of different serological methods and the difference in sensitivity may also be responsible for the divergences^30^.

Most infections were past or latent. The present results agree with those obtained in Cameroon by Ayeah et al.^30^, who found that majority of infections are often past infections (72.7%) with few cases of *Toxoplasma*-specific IgM (1.3%). In Iran, Babaie et al. 12 noted that the seroprevalence of *Toxoplasma* IgG antibodies in pregnant women was 34.4% while 18.8% out of IgG-positive women showed specific IgM antibodies. Furthermore, in meta-analysis published by Foroutan-Rad et al.^34^, the seroprevalence rate of latent and acute toxoplasmosis during pregnancy was calculated as 38% and 4%. According to Many and Koren^35^, the high seroprevalence of anti-*T. gondii* IgG may indicate that most women got infected possibly 6 to 12 months before pregnancy. IgM antibodies are the first class of antibodies detected after a primary *Toxoplasma* infection and they can persist a long time in the serum^36^. However, they also decline very rapidly than IgG antibodies and this is probably why most *Toxoplasma* infections detected are IgG.

### Risk factors associated with *T.gondii* infection

Concerning the area of residence, Flatt and Shetty 10 have found similar results with the current study. However, Silva-Díaz et al.^25^ have found higher seroprevalences in urban areas. A significant number of rural residents, inadequate health literacy, and restricted accessibility to health care facilities. Observed result regarding habit of eating raw vegetables was in agreement with the study of Negero et al.^29^. Insufficient hygienic method for transportation and selling of vegetables together with the poor quality water for washing vegetables might have contributions for the contamination of vegetables by *T.gondii* oocysts. The contamination with *T.gondii* was more likely observed in those participants who consumed tap water. This indicates that contamination of tap water by *T.gondii* oocysts from felids feces indicate inadequate water management. This is in consistent with previous report of Negero et al.^29^ and Soltani et al.^14^. Soil contact was highly associated with the infection. This result agrees with different findings^12^,^13^ and oppose with some previous studies of 10,26,29. We found gardening and farm work as a risk factor because of the rural nature of a large proportion of Ain Defla (flats that have access to gardens). When it comes to the possibility of contracting *T.gondii* infection, the most dangerous risk factors are eating raw or undercooked meat, having close contact with cats and soil, and engaging in inadequate hygiene standards. Taking the example of undercooked meat as a main source of the infection, this eventuality is very rare in Algeria due to the country's culture, religion and different meat-eating habits, thorough cooking is always preferred. However, in another study conducted in Algeria by Messerer et al.^37^, major risk factors were consumption of poorly-cooked meat and exposure to cats contradicting our study. The importance of the current study is that it is one of the few – to our knowledge – pregnant women population based studies performed in Algeria. However, there are some restrictions. Firstly, only seroprevalence was applied to detect positive people and the predominant strain of *T. gondii* were unknown. Secondly, the serological survey represent a pretty low number of population, other studies must be carried out to determine the true prevalence of *T. gondii* infection in pregnant women in different regions of Algeria.

## Conclusions

The results highlight the need to raise awareness of *T. gondii* infection, specifically with regard to the way infections occur so that women can take steps to protect themselves and avoid contracting with this pathogenic parasite. It is imperative that educational programs be mounted to create awareness among the public, as well as among health personnel, and to work towards prenatal screening for toxoplasmosis.

## Figures and Tables

**Figure 1:**
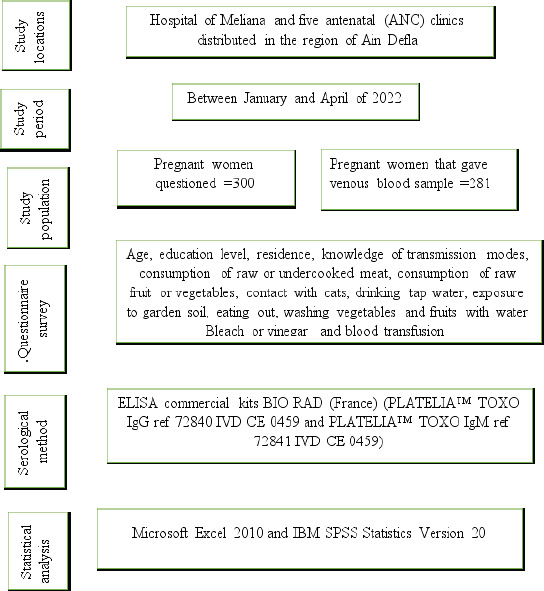
An infographic of the overall methodology

**Table 4:** Multivariate analyses for factors associated with *T.gondii* infection

	A	E.S.	Wald	Ddl	p.	OR	IC for OR 95%	

							Lower	Upper
Consumption of undercooked meat (Yes)	-1.237	0.501	6.095	1	0.014	0.290	0.109	0.775
Consumption of raw vegetables (Yes)	0.653	0.305	4.569	1	0.033	1.921	1.056	3.495
Consumption of water other than tap water (Yes)	0.933	0.296	9.924	1	0.002	2.543	1.423	4.546
Contact with soil (Yes)	1.360	0.374	13.238	1	0.000	3.894	1.872	8.100
Meals at home (No)	0.763	0.320	5.671	1	0.017	2.144	1.145	4.018
Constante	-1.956	0.284	47.558	1	0.000	0.141		
